# The impact of elbow and knee joint lesions on abnormal gait and posture of sows

**DOI:** 10.1186/1751-0147-50-5

**Published:** 2008-02-28

**Authors:** Rikke K Kirk, Bente Jørgensen, Henrik E Jensen

**Affiliations:** 1 Department of Veterinary Pathobiology, Faculty of Life Sciences, University of Copenhagen, Denmark; 2 Danish Institute of Agricultural Sciences, Research Centre Foulum, Tjele, Denmark; 3 Novo Nordisk A/S, Novo Nordisk Park, 2760 Maaloev, Denmark

## Abstract

**Background:**

Joint lesions occur widespread in the Danish sow population and they are the most frequent cause for euthanasia. Clinically, it is generally impossible to differentiate between various types of non-inflammatory joint lesions. Consequently, it is often necessary to perform a post mortem examination in order to diagnose these lesions. A study was performed in order to examine the relation of abnormal gait and posture in sows with specific joint lesions, and thereby obtaining a clinical diagnostic tool, to be used by farmers and veterinarians for the evaluation of sows with joint problems.

**Methods:**

The gait, posture and lesions in elbow- and knee joints of 60 randomly selected sows from one herd were scored clinically and pathologically. Associations between the scorings were estimated.

**Results:**

The variables 'fore- and hind legs turned out' and 'stiff in front and rear' were associated with lesions in the elbow joint, and the variables 'hind legs turned out' and 'stiff in rear' were associated with lesions in the knee joint.

**Conclusion:**

It was shown that specified gait and posture variables reflected certain joint lesions. However, further studies are needed to strengthen and optimize the diagnostic tool.

## Background

Joint lesions are a major cause of euthanasia and culling of sows in Denmark and are of importance both economically and in relation to animal welfare [1]. Joint lesions of sows are frequent causes of leg weakness, and non-inflammatory joint diseases as arthrosis and osteochondrosis are main causes of lameness [2-4]. Osteochondrosis developes in growing animal and is due to a failure in the endochondrale ossification of the articular cartilage and the growth plate [5]. The lesions caused by osteochondrosis can heal completely [2] or progress into secondary arthrosis in the adolescent animal [5]. The aetiology of osteochondrosis is thought to be multifactorial, and trauma, heredity, rapid growth, nutrition, and anatomical conformation are factors associated with this disease [5-7]. Non-osteochondrosis-related arthrosis (i.e. primary arthrosis) is characterized by fibrillation and ulceration of the articular cartilage and of eburnation of the subchondral bone [5]. The pathogenesis of primary arthrosis of sows is not well understood, but the confinement of sows and the subsequent limitations of exercise have been suggested as a possible aetiology [8]. Osteochondrosis and arthrosis in sows are often bilateral and symmetrical and are frequently observed in the distal humerus and femur [2].

Focus on the association between clinical observations and lesions of the locomotive system has been the objective in only a few porcine studies [3,4]. Therefore, it is uncertain which specific joint lesions actually are associated with the different types of abnormal gait and posture in pigs. The clinical examination of sows has until now been of limited use when trying to asses the cause of lameness, and a post-mortem examination of the animal has been preferred to differentiate the various causes of lameness [3].

The present study was performed in order to examine the correlations between certain joint lesions and defined gait and posture variables in sows.

## Methods

### Animals and housing

An observational prospective study was carried out in a Danish pig herd. Sixty randomly selected crossbred Landrace-Yorkshire (LY) sows from the herd were included. The sows were tethered during the gestation, with concrete floor in the lying area, and slatted floor in the dunging area. The farmer decided exclusively when to cull the sows, which did not differ from usual procedures. The time of culling was recorded and varied from first to ninth parity.

### Gait and posture scoring

The gaits and postures variables, which were often bilateral, were scored before first mating and after every farrowing until culling. The variables were defined according to earlier publications [9,10]. The scoring procedure was performed by one observer outside the pen with the animal in motion. The following 11 variables of the gait and posture, of which buck-kneed forelegs, fore and hind legs turned out, and stiff in front and rear have been shown to be associated with osteochondrosis and arthrosis [9], were scored on a scale from 1 (normal) to 5 (severe):

• Buck-kneed forelegs

• Forelegs turned outwards

• Upright pasterns forelegs

• Weak pasterns forelegs

• Standing under position hind legs

• Hind legs turned outwards

• Steep hock joint

• Weak pasterns hind legs

• Stiff in front

• Stiff in rear

• Swaying hindquarters

### Pathology

Elbow and knee joints were collected at slaughter. Complete sets of joints were obtained from 33 animals, while incomplete sets were sampled from 27 sows. In these cases the following materials were missing: left radius and ulna (one sow); right elbow joint (9 sows); left (20 sows) and right (24 sows) knee joint.

**Figure 1:**
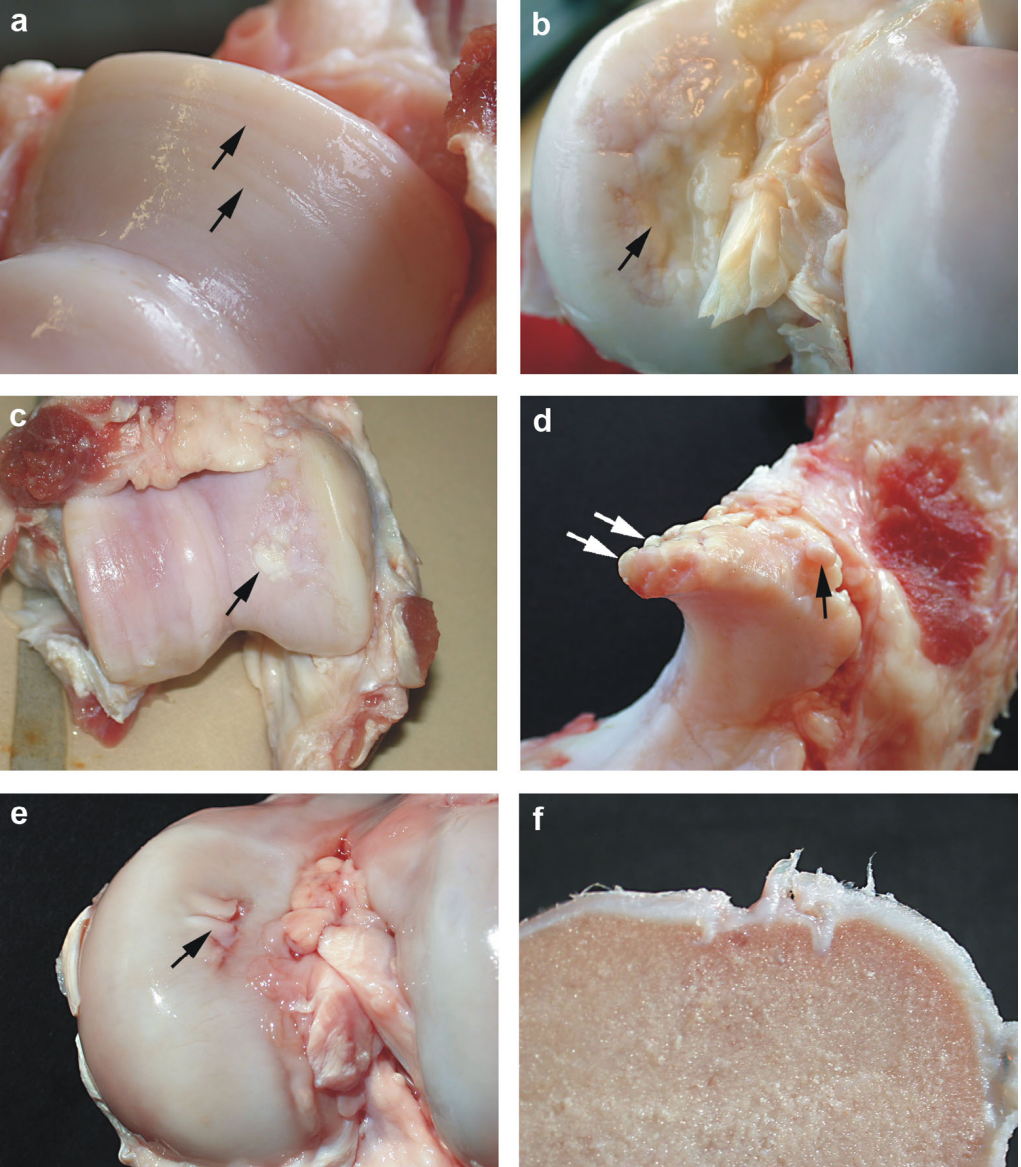
**Template for categorizing macroscopical joint lesions in sows**. a: Cartilage erosion (arrows) on the medial humeral condyle. b: Cartilage ulceration (arrow) on the medial femoral condyle. c: Cartilage repair (arrow) of the medial femoral condyle d: Marginal osteophytes (arrows) on processus anconeus of ulna. e: Cartilage infoldings (arrow) on the medial femoral condyle. f: Cartilage infoldings on the medial femoral condyle. Cross section of Fig. 2e.

All joints were opened and evaluated macroscopically in specified locations: (I) the medial humeral condyle, (II) the lateral humeral condyle, (III) fovea capitis radii, (IV) incisura trochlearis of ulna, (V) processus anconeus of ulna, (VI) the medial femoral condyle, and (VII) the lateral femoral condyle. The locations were assesed for the presence of: (a) erosions, (b) ulcerations, (c) repair reactions, (d) marginal osteophytes, and (e) infolding of the joint cartilage according to a template (Fig. 1a–f) and scored as normal (0), moderate (1), when the lesion involved less than 20% of the articular surface or severe (2), when the lesion exceeded 20% of the articular surface.

**Figure 2:**
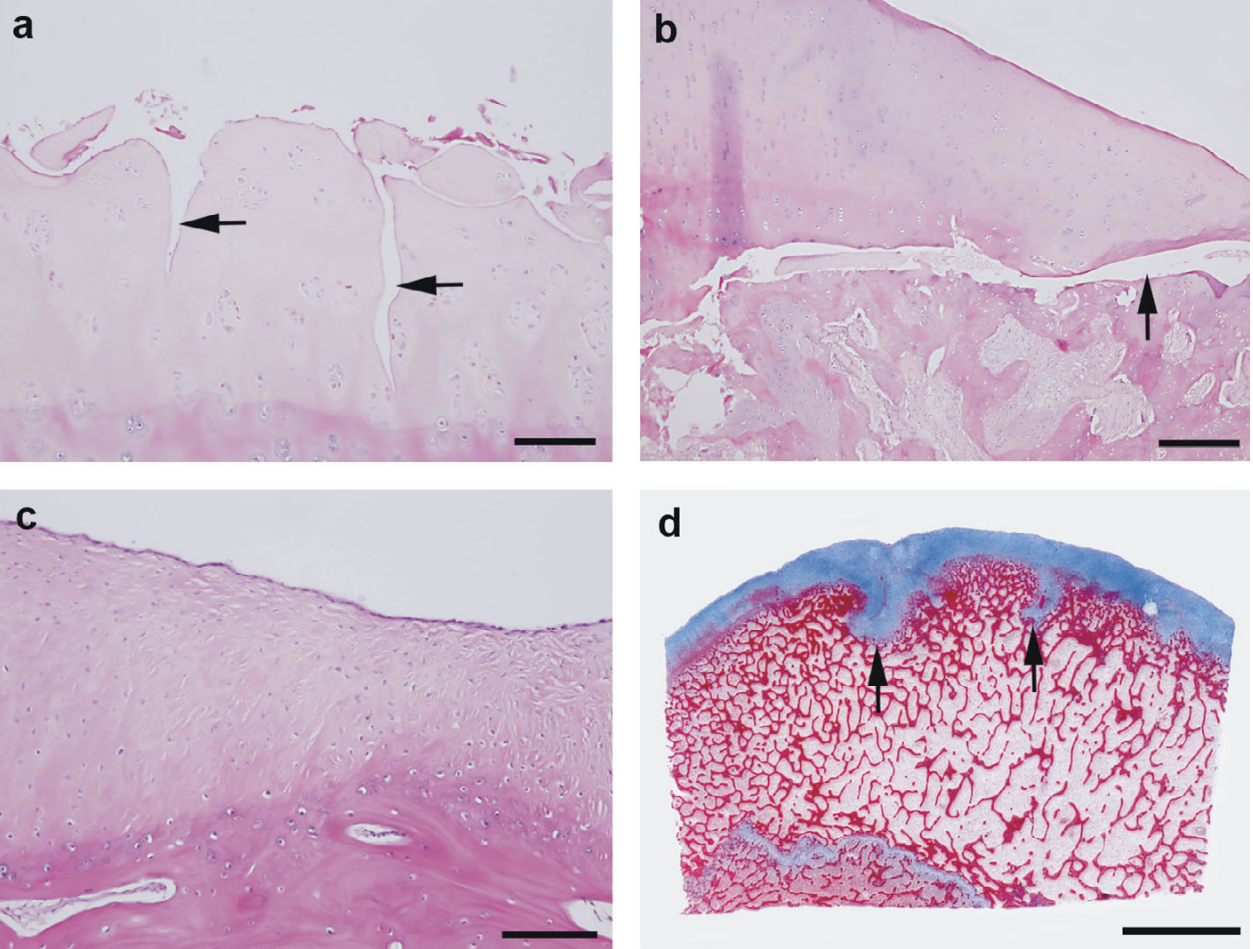
**Template for histological classification of joint lesions in sows**. a: Superficial cartilage erosions (arrows) of variable thickness are present. Articular cartilage of the medial humeral condyle. Haematoxylin and eosin. Bar = 100 μm. b: Typical osteocondrotic lesion in the form of osteochondritis dissecans (arrow). Articular cartilage of the lateral humeral condyle. Haematoxylin and eosin. Bar = 200 μm. c: Fibrous tissue and fibrocartilage are filling out a defect of the articular cartilage. Articular cartilage of the medial humeral condyle. Haematoxylin and eosin. Bar = 125 μm. d: Infoldings of thickened (retained) articular cartilage are present (arrows). Articular cartilage and subchondral bone of the medial humeral condyle. Masson's Trichrome. Bar = 5 mm.

In order to confirm the nature of the macroscopical lesions, a representative number of the specified joint lesions was evaluated histologically according to a template (Fig. 2a–d) and according to the following definitions: (I) erosion: thinning and loss of the surface cartilage, (II) ulceration: the articular cartilage was lost and the subchondral bone was exposed, including flap formation in osteochondritis dissecans lesions, (III) repair: defect in the cartilage substituted by fibrous tissue or fibrocartilage, (IV) osteophytes: formation outside the bone consisting of osseous trabeculae, and (V) infolding: articular cartilage was protruding into the subchondral bone.

### Statistical methods

The PROC CORR procedure in SAS was used for analysis, and the mutual correlations between similar lesions of left and right side were analysed. The same procedure was used for analysing the mutual correlation between lesions in the same joint, one side at a time.

The frequencies of scorings of the gait and posture variables were analysed. The associations between the gait and posture variables and the joint lesions were analysed one at a time by using the maximum score over time of the 11 variables for each sow against all the joint lesion scores. A backward elimination procedure was used by removing one variable at a time with highest *P*-value until only variables with a *P*-value below 0.5 were left in the model. The frequencies of the joint lesions were examined and lesions observed in less than 10% of the animals were eliminated from the analyses. The procedure PROC GLM in SAS was used to estimate Pearson correlation coefficients. [11]

## Results

Joint lesions were observed more often in the elbow joint compared to the knee joint (Tables 1 and 2). The most frequent lesion in the elbow joint was erosion of the articular cartilage, in particular on the medial humeral condyle (left side 95%, and right side 84%). Also ulceration (left side 18%, right side 10%) and repair (left side 13%, right side 16%) of the articular cartilage of the medial humeral condyle, as well as formation of marginal osteophytes of processus anconeus (left side 14%, right side 16%) of ulna were often observed. In the knee joint, erosion (left side 15%, right side 42%) and ulceration (left side 10%, right side 6%) of the articular cartilage of the medial femoral condyle were noted as the most frequent lesions.

**Table 1:** Number of certain lesions in left and right elbow of 60 sows.

**Score**	**Humerus**										**Radius**						**Ulna**					
	*Medial condyle*						*Lateral condyle*				*Fovea capitis*						*Incisura trochlearis*				*Proc. anconeus*	
	Erosion		Ulceration		Repair		Erosion		Ulceration		Erosion		Ulceration		Osteophytes		Erosion		Ulceration		Osteophytes	
	L	R	L	R	L	R	L	R	L	R	L	R	L	R	L	R	L	R	L	R	L	R
0	3	8	49	46	52	43	25	15	56	50	30	32	59	51	55	50	43	38	59	51	51	43
1	31	27	8	3	7	5	29	27	3	1	26	17	0	0	1	1	13	11	0	0	3	5
2	26	16	3	2	1	3	6	9	1	0	3	2	0	0	3	0	3	2	0	0	5	3

Score: 0 = no lesion; 1 = moderate lesion; 2 = severe lesion. L = left side; R = right side.

**Table 2:** Number of certain lesions in left and right knee joints of 60 sows.

**Score**	**Femur**											
	*Medial condyle*								*Lateral condyle*			
	Erosion		Ulceration		Repair		Infolding		Erosion		Ulceration	
	L	R	L	R	L	R	L	R	L	R	L	R
0	34	30	36	34	38	33	38	30	38	36	38	34
1	5	4	0	1	0	3	2	4	0	0	1	2
2	1	2	4	1	2	0	0	2	2	0	1	0

Score: 0 = no lesion; 1 = moderate lesion; 2 = severe lesion. L = left side; R = right side

Because a significant correlation between similar lesions of the left and the right side (from r = 0.25 to r = 0.71) was found, the two sides were subsequently pooled.

The mutual correlations between lesions within the joints (Tables 3 and 4) showed a strong correlation between erosions in the lateral condyle of humerus and cartilage erosion of incisura trochlearis on ulna *(P *< 0.001) and between erosions in the lateral condyle of humerus and marginal osteophytes on the processus anconeus of ulna (*P *< 0.001). However, no correlations were seen between the same types of lesions in the medial condyle of humerus. Also a strong correlation between cartilage erosion of fovea capitis on radius and cartilage erosion of incisura trochlearis on ulna was observed (*P *< 0.001). In the knee joints a strong correlation between erosion and ulceration in the medial condyle of femur was registered (*P *< 0.01).

**Table 3:** Correlation (r) between joint lesions within the elbow joint.

			**Humerus**						**Radius**		**Ulna**			
			*Medial condyle*				*Lateral condyle*		*Fovea capitis*		*Incisura trochlearis*		*Processus anconeus*	
			Ulceration		Repair		Erosion		Erosion		Erosion		Osteophyt	
			L	R	L	R	L	R	L	R	L	R	L	R
**Humerus**	*Medial condyle*	Erosion	0.31*	0.13	0.18	0.23	0.01	0.17	0.22	-0.02	0.13	-0.07	0.25	0.23
		Ulceration			-0.08	0.29*	0.12	-0.01	0.05	0.17	0.10	-0.09	0.17	-0.12
		Repair					-0.14	0.07	0.05	0.16	0.26	0.05	0.09	-0.03
	*Lateral condyle*	Erosion							-0.01	0.18	0.27*	0.53***	0.27*	0.50***
**Radius**	*Fovea capitis*	Erosion									0.44***	0.44***	0.14	0.10
**Ulna**	*Incisura trochlearis*	Erosion											0.40**	0.19

No. of sows = 60. L = left side; R = right side. Levels of significance: * *P *≤ 0.05; ** *P *≤ 0.01; ***P ≤ 0.001.

**Table 4:** Correlation (r) between joint lesions within the knee joint. .

			**Femur**			
			*Medial condyle*			
			Ulceration		Infolding	
			L	R	L	R
**Femur**	*Medial condyle*	Erosion	0.43**	NR	NR	-0.17

No. of sows = 60. L = left side; R = right side; NR = Not registered. Levels of significance: ** *P *≤ 0.01

**Figure 3:**
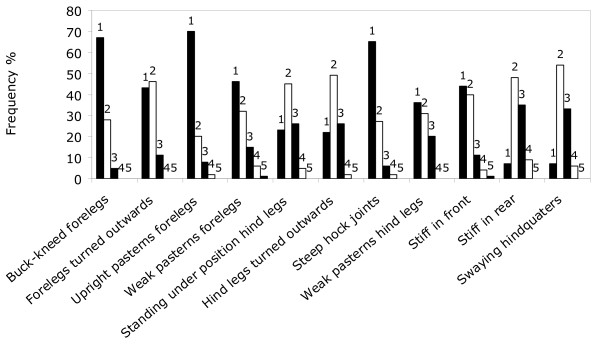
**Frequency distribution of scorings (from 2 to 9 times/sow) of gait and posture variables in 60 sows**. Score from 1 (normal) to 5 (severe).

The scorings of the variable 'stiff in rear' and 'swaying hindquarters' showed that 44% and 39% of scorings, respectively, were between 3 and 5 (Fig. 3).

The highest degree of positive associations were between 'hind legs turned out' and repair of the articular cartilage of the medial femoral condyle (*P *< 0.001) and with marginal osteophytes of the fovea capitis on radius (*P *< 0.01), and 'weak pasterns forelegs' and with marginal osteophytes of the fovea capitis on radius (*P *< 0.001) (Tables 5 and 6). 'Forelegs turned out' were positively associated with erosions of incisura trochlearis on ulna (*P *< 0.05). Moreover, significantly positive associations between 'stiff in front and in rear' and ulceration of the cartilage of the lateral humeral condyle were observed (*P *< 0.05). Significantly negative associations were found between 'weak pasterns on forelegs' and cartilage ulceration of the medial humeral condyle (*P *< 0.05) and cartilage infoldings of the medial femoral condyle that were verified to be of osteochondrotic origin (*P *< 0.01). A negative association was also found between 'stiff in rear' and cartilage erosion of radius (*P *< 0.05) and cartilage ulceration of the medial femoral condyle (*P *< 0.01).

**Table 5:** Association (regression coefficients) between gait, posture (the maximal scores over time for each sow were used) and lesions in the elbow joint. .

	**Humerus**			**Radius**		**Ulna**
	*Mediale condyle*		*Lateral condyle*	*Fovea capitis*		*Incisura troichlearis*
	Ulceration	Repair	Ulceration	Osteophytes	Erosion	Erosion
Forelegs turned out						0.22*
Hind legs turned out				1.74**		
Weak pasterns forelegs	-0.62*			3.16***		
Stiff in front			0.85*			
Stiff in rear			0.87*		-0.33*	

No. of sows = 60. Levels of significance: * *P *≤ 0.05; ** *P *≤ 0.01; *** *P *≤ 0.001

**Table 6:** Association (regression coefficients) between gait, posture (the maximal scores over time for each sow were used) and lesions in the knee joint.

	**Femur**			
	*Medial condyle*			*Lateral condyle*
	Ulceration	Repair	Infolding	Ulceration
Weak pasterns forelegs	0.58*		-0.90**	
Hind legs turned out		1.12***	-0.55*	
Stiff in rear	-0.73**			1.00**

No. of sows = 60. Levels of significance: * *P *≤ 0.05; ** *P *≤ 0.01; *** *P *≤ 0.001.

Associations to the first and the last scoring were examined, too, but did not influence the results. No significant effect of parity was found.

## Discussion

Correlations between various lesions on the same articular surfaces and between lesions of opposing articular surfaces in the elbow and knee joints were observed. It was not obvious from the correlations which types of lesions preceded the other ones. However, because histology revealed erosions of the articular cartilage without ulcerations (Fig. 2a), it was most likely that erosions preceded ulcerations. An exception from this was in cases of osteochondritis dissecans, where ulceration was seen without erosion being present (Fig. 2b).

In accordance with results obtained in a previous study [4], a correlation between erosion in the articular cartilage of the lateral humeral condyle and the presence of marginal osteophytes on processus anconeus of ulna was observed. The presence of marginal osteophytes was always observed together with erosion of the articular cartilage. By contrast, erosions were often seen without marginal osteophytes. Therefore, it is likely that cartilage lesions precede the formation of marginal osteophytes. However, in humans osteophytes may be present without any affection of the cartilage [12], and it is assumed to be an adaptive and stabilizing reaction caused by instability of joints [13]. Therefore, it could be speculated that both cartilage lesions and osteophytes in sows are caused by joint instability.

The positive association between forelegs that are turned out and stiff movements of the front and rear legs and cartilage lesions in the elbow joint is in agreement with the results obtained by Jørgensen [9]. Weak pasterns on forelegs were both negatively and positively associated with lesions in the elbow and knee joints, but in particular marginal osteophytes on radius were positively associated (*P *< 0.001). The presence of weak pasterns on forelegs has previously been found to be positively associated with normal, brisk gait and negatively associated with osteochondrosis/osteoarthrosis [9]. However, in contrast to a study by Jørgensen (9), in which only lesions in the knee joint had an impact on hind legs being turned out, it was found that also lesions in the elbow joint were associated with this abnormal posture.

## Conclusion

In the present study it was shown that some defined gait and posture variables reflected specific joint lesions in sows. Presence of 'stiff in front and rear legs' and 'forelegs turned out' were highly indicative of osteochondrotic and arthrotic lesions in the elbow joint. These observations could be helpful in the selection procedure of breeding animals and should encourage farmers to include animals with a low incidence of osteochondrosis in breeding programmes. However, further studies are needed to further strengthen and optimize the diagnostic tool.

Moreover, it was found that correlations between certain articular lesions exist.

## Competing interests

The author(s) declare that they have no competing interests.

## Authors' contributions

BJ designed the study and developed the gait and posture scoring methods. RKK examined and scored the joint lesions with assistance from HEJ. RKK and BJ performed the statistical analysis and RKK, BJ, and HEJ drafted the manuscript. All authors read and approved the final manuscript.
